# Accordance to the Dietary Approaches to Stop Hypertension diet pattern and cardiovascular disease in a British, population-based cohort

**DOI:** 10.1007/s10654-017-0354-8

**Published:** 2018-01-09

**Authors:** Nicholas R. V. Jones, Nita G. Forouhi, Kay-Tee Khaw, Nicholas J. Wareham, Pablo Monsivais

**Affiliations:** 1 UKCRC Centre for Diet and Activity Research (CEDAR) and Medical Research Council (MRC) Epidemiology Unit, University of Cambridge School of Clinical Medicine, Box 285 Institute of Metabolic Science, Cambridge Biomedical Campus, Cambridge, CB2 0QQ UK; 2 Department of Public Health and Primary Care, University of Cambridge School of Clinical Medicine, Cambridge, UK; 3 Department of Nutrition and Exercise Physiology, Washington State University Elson S Floyd College of Medicine, Spokane, 99210 USA

**Keywords:** DASH, Cardiovascular, Stroke, Food, Diet, Prevention, ICD-10

## Abstract

The dietary approaches to stop hypertension (DASH) diet could be an important population-level strategy to reduce cardiovascular disease (CVD) in the UK, but there is little UK-based evidence on this diet pattern in relation to CVD risk. We tested whether dietary accordance with DASH was associated with risk of CVD in a population-based sample of 23,655 UK adults. This prospective analysis of the EPIC-Norfolk cohort study analysed dietary intake (assessed using a validated food frequency questionnaire) to measure accordance with DASH, based on intakes of eight food groups and nutrients, ranking the sample into quintiles. Cox proportional hazards regression models tested for association between DASH accordance and incident stroke, ischemic heart disease (IHD) and total incident CVD (stroke and IHD only), as well as CVD mortality, non-CVD mortality and total mortality. Hazard ratios (HR) and 95% confidence intervals (CI) were estimated adjusting for age, sex, behavioral and clinical risk factors and socioeconomic status. Over an average of 12.4 years follow-up, we ascertained 4129 incident CVD events, of which stroke accounted for 1011. Compared to participants with the least DASH-accordant diets, those with the most DASH-accordant diets had 20% lower risk of incident stroke (HR, 95% CI 0.80, 0.65–0.99) and 13% lower risk of total incident CVD (0.88, 0.79–0.99) but no lower risk of CHD (0.90, 0.79–1.02). CVD-related mortality also showed strong inverse associations with DASH accordance (0.72, 0.60–0.85). This study provides evidence for the cardioprotective effects of DASH diet in a UK context.

## Introduction

Cardiovascular disease (CVD) is the most common cause of death in the world and was estimated to account for 32% of all deaths in 2013 [1]. CVD is also a major concern for the United Kingdom (UK) in particular, with the 2010 Global Burden of Disease Study showing that the leading cause of years of life lost in the UK was Ischaemic Heart Disease (IHD), followed by stroke as the third main cause [2]. A 2012 review found that CVD was responsible for 42,000 premature deaths (defined as deaths before the age of 75) in the UK and that it was the most common cause of death in women [3]. In addition to the cost to life, the health burden attributable to CVD imposes costs on the healthcare system and the wider economy, with these costs estimated to have been £29.1 billion in 2004 [4].

The importance of dietary risk factors in causing CVD has been broadly accepted, with dietary modification now considered an integral component of a comprehensive, population-level CVD prevention strategy [5]. The importance ascribed to individual nutrients in preventing CVD has been diminished, replaced by an emphasis on foods and overall dietary patterns [6]. In two long term trials, a diet based around nutrient targets—specifically reduced fat intake—was found to be less effective at reducing risk of CVD events than a dietary pattern based around changing the consumption of whole foods [6–8]. Those diet patterns found to reduce CVD risk have been focused on increased consumption of fruit, vegetables, whole grains, fish, nuts, dairy products and vegetable oils, and reduced consumption of processed meats, sugars and desserts, alcohol and fats [9].

The dietary approaches to stop hypertension dietary pattern (or ‘DASH diet’) was developed in the 1990s and has many of the characteristics of a typical cardioprotective diet; it was first used in a controlled feeding trial, where it was found to be effective at reducing blood pressure [10]. The effectiveness of the DASH diet for reducing blood pressure and other cardiovascular risk factors was further supported by subsequent experimental evidence [11–16]. Observational studies have found an association between accordance to the DASH diet and cardiovascular disease outcomes, both fatal and non-fatal, in studies from the USA, Italy and Sweden [17, 18]. However, to date no studies examining this relationship have been conducted in the UK and as such the effectiveness of the pattern at reducing CVD risk in the UK population is unknown.

Demonstrating the potential effectiveness of the DASH diet in different contexts is important, given that prospective epidemiological studies have typically characterised diets based on a relative measure of accordance with the DASH dietary pattern [19–21]. For example, absolute intakes of some dietary components that have been used to assess DASH accordance, including milk, fruit juice, sugar-sweetened beverages and red meat, vary substantially among countries [22, 23]. Other important components of the diet differ too. For example, while sugar-sweetened beverages are the leading source of added sugars in the USA [24], table sugar, preserves, confectionary and grain-based foods are more important sources in the UK and other European countries [25]. Studying the DASH-CVD relationship in a UK cohort both adds to the international evidence base and provides UK-specific evidence that may be particularly valuable if the DASH diet were ever to be considered as a possible exemplar diet for use in UK public health messaging.

This study examined the relationship between the DASH dietary pattern, incident CVD and CVD mortality in a prospective cohort from the East of England, with the aim of determining whether or not accordance with this dietary pattern is associated with risk of CVD events in the UK. The hypothesis was that increased accordance to the DASH diet would be associated with lower risk of incident CVD and CVD mortality.

## Methods

### Subjects

**Fig. 1:**
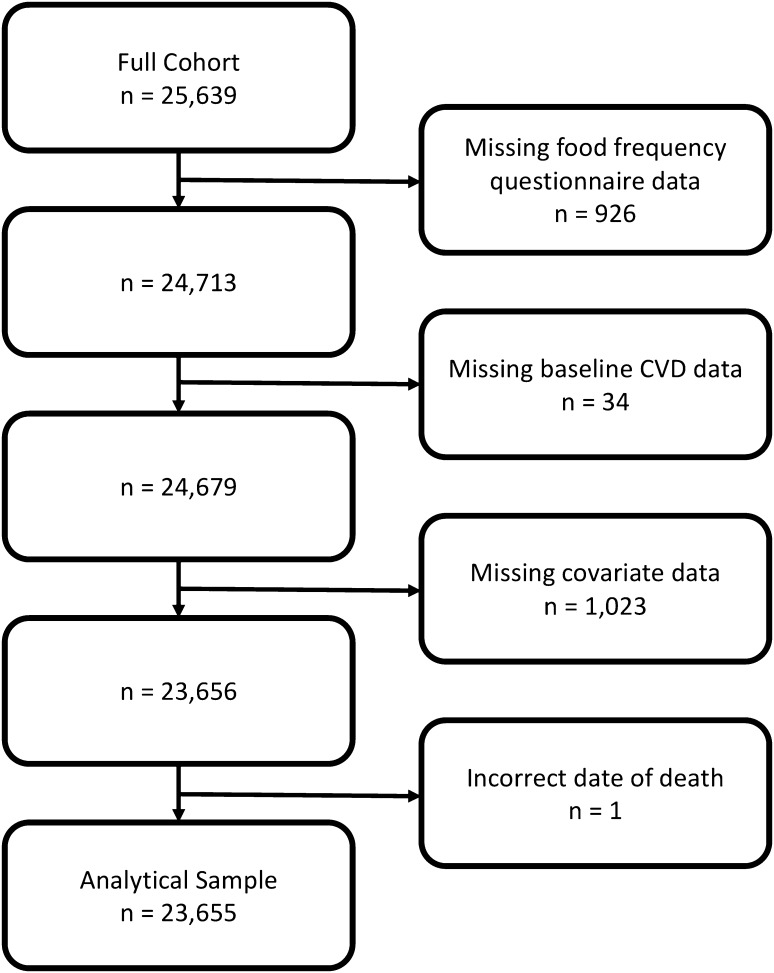
Flow diagram illustrating selection of the analytical sample

This study used data from participants who, at baseline, were aged between 39 and 79 years and lived in the East of England. The participants were members of the EPIC-Norfolk cohort study, which contains 25,639 people. Of these, 926 people were excluded because they did not have a completed FFQ, 34 people with missing data about baseline cardiovascular disease and diabetes, 1023 people with missing data in one or more covariate required in these analyses, and 1 person with a date of death recorded prior to their first health check, resulting in an analytical sample of 23,655. For analyses where the outcome studied was required to be an incident event we also excluded 863 people with self-reported IHD and 325 people with self-reported stroke at baseline, as necessary for the outcome. Figure 1 presents a flowchart showing how the analytical sample was reached and Table 1 compares the analytical sample and the excluded cases.

**Table 1:** Comparison of analytical sample and excluded cases

	Included (n = 23,655)	Excluded (n = 1984)
Mean age (years)	59.1	61.3
% Male	45.6	40.9
% Current smoker	11.5	15.1
Mean BMI	26.3	26.9
% Inactive^a^	29.7	42.9
Mean weekly units of alcohol	7.2	6.2
Semi- and non-skilled occupational social class^b^	16.5	24.6
% Single	3.9	6.1
% Diabetes^c^	2.3	2.7
% Anti-hypertensive medication^d^	18.5	22.5
% Lipid lowering medication^e^	1.5	1.4
Mean total serum cholesterol (mmol/L)	6.2	6.3
Mean LDL cholesterol (mmol/L)	3.9	4.0
Mean HDL cholesterol (mmol/L)	1.4	1.4
Mean systolic blood pressure (mmHg)	135.3	137.4
Mean diastolic blood pressure (mmHg)	82.5	83.3
% Least accordant DASH quintile	24.3	26.7

^a^Lowest category of physical activity; ^b^lowest two occupational social groups, based on the Registrar General classification; ^c^self-reported past diagnosis of diabetes; ^d^use of any medications indicated for hypertension; ^e^use of any medications indicated for high cholesterol or dislipidemia

The EPIC-Norfolk study is part of a Europe-wide initiative initially developed to investigate the relationship between diet and cancer risk, which has since developed to include other clinical endpoints in addition to cancers [26]. Participants were recruited from the population aged between 39 and 79 years registered with general practices in the city of Norwich, its surrounding towns, and rural areas in Norfolk between the years 1993 and 1999. Those who were contacted and gave consent were invited to a health check, at which measurements and samples were taken for height, weight, body circumferences, blood pressure, urine and respiratory function. Dietary data were collected using a food frequency questionnaire (FFQ), described below, and other data were collected using a questionnaire on cigarette and alcohol consumption, physical activity, socioeconomic status (SES) and history of cardiovascular disease. The total EPIC-Norfolk cohort was found to be similar to the wider English population in 1993 in terms of height, weight, BMI, systolic blood pressure and total cholesterol [26]. Further details about the EPIC-Norfolk cohort have been published by Day et al. [26].

### Dietary assessment

The typical food intake of study participants was assessed using a food frequency questionnaire adapted from an FFQ originally used in the Nurse’s Health Study, which has been adapted and validated for use in the UK [27]. This FFQ contains 130 line items and asks participants about the frequency at which they consumed each item across the previous year, from never or less than once per month to six or more times per day. Full details about this FFQ and how it was developed have been published previously by Welch et al. [28]. Estimated intake of each food or food group consumed was calculated using the FETA program, which combines the frequency of consumption reported in the FFQ with standardised portion data to estimate the intake of macro- and micro-nutrients [29]. FETA has been described previously in detail by Mulligan et al. [29].

### Exposure: DASH accordance score

The DASH accordance score was developed using the method set out by Fung et al. [19], which has been compared to three other methods for calculating accordance with the DASH diet pattern and found to produce similar results when measuring the association between DASH and colorectal cancer [20]. This method is based on an individual’s consumption of six food groups and two nutrients. Of these, five—fruit, vegetables, nuts and legumes, whole grains, and low-fat dairy—are encouraged and three—red and processed meats, salt, and non-milk extrinsic sugars (NMES)—are discouraged.

The energy-adjusted consumption of each of these groups was calculated using the residual method, previously described by Willett [30]. For the five encouraged food groups the quintile is the individual’s score for that group, I.E., those who consume the most are in quintile five and would have a score of five (the highest score), and for the three discouraged groups this is inverted so that the greatest consumers have a score of one. The quintile scores for all eight groups are summed to produce a measure of DASH accordance that has a potential range of 8–40, with higher scores indicating that the individual’s diet is in greater accordance with the DASH diet pattern. This score is then divided into five quintiles (range in this study: 9–19, 20–23, 24–25, 26–29, 30–38), so that Quintile 5 contained those people with the most DASH-accordant diets. This method for assessing accordance with the DASH diet pattern has been used previously in the EPIC-Norfolk cohort and details of the foods contributing to each group are reported in a study by Monsivais et al. [31].

### Outcomes: incident CVD and CVD mortality

The EPIC study collects data on incident events and mortality through linkage to hospital admissions and death certificates, which has been described in detail by Lachman et al. [32]. Follow-up for incident events continued until 31st March 2009 and until 31st December 2013 for mortality.

The outcomes of interest were incident CVD and CVD mortality, looking at the following specific outcomes [International Classification of Disease (ICD-10) codes in brackets where appropriate]: (1) incident Ischaemic heart disease (IHD) (I20–25), (2) incident cerebrovascular disease (stroke) (I60–69), (3) total incident CVD (I20–25, I60–69), (4) total CVD mortality (I20–25, I60–69), (5) non-CVD mortality, and (6) all-cause mortality. All-cause and non-CVD mortality were examined because they can reveal whether any association between DASH and health outcomes is specific to CVD or is associated with other causes of death, which may indicate residual confounding.

### Other covariates

In order to address potential confounding, we used multivariable models to adjust for total dietary energy (continuous), sex (male, female), age in years (continuous), smoking (current smoker, former smoker, never smoked), body mass index (continuous), physical activity (inactive, moderately inactive, moderately active, active), alcohol consumption (mean units per day), SES (based on occupation, according to the Registrar General’s classificaiton scheme: non-skilled, semi-skilled, skilled—manual, skilled—non-manual, manager, professional), marital status (single, married, widowed, separated, divorced), diabetes (yes, no), presence of antihypertensive medication (yes, no), presence of lipid-lowering medication (yes, no), and previous history of cardiovascular disease (yes, no) (although in models where an incident case of that disease was the outcome these people were excluded). Although presumed to be associated with DASH accordance and CVD outcomes, blood pressure and blood lipids were not included in the main models because they lie on the proposed causal pathway between DASH accordance and CVD, although they were included in a sensitivity analysis. Physical activity was measured using the EPAQ2 questionnaire, which aimed to capture physical activity across multiple domains to give a measure of overall activity [26]. This questionnaire has been validated against objectively measured physical activity and found to be acceptable for this use [33].

### Statistical analysis

Cox proportional hazards models were fitted to calculate the hazard ratio (HR) and a 95% confidence interval for the relationship between quintile of DASH accordance and the outcomes studied, using the quintile of least accordance (quintile 1) was the reference category. For each outcome of interest two models were produced: (1) a model adjusted for age, sex and total dietary energy; (2) as Model 1 but further adjusted for smoking status, physical activity, alcohol intake, diabetes, BMI, SES, presence of antihypertensive medication, presence of lipid-lowering medication, and previous history of cardiovascular disease. We also calculated the absolute rate for each disease to enable comparisons with studies conducted in other populations. Time at risk began from the date of the first health check and ended at date of death or date of diagnosis, as appropriate, or was right-censored by the end of follow-up.

Two sets of sensitivity analyses were also conducted. One of these was as Model 2, above, but also included total serum cholesterol and systolic blood pressure. The other used a continuous rather than categorical measure of DASH accordance.

All analyses were conducted in *Stata SE 13.1* [34].

## Results

Data from 23,655 people present in the EPIC-Norfolk cohort were analysed to explore the difference in risk across quintiles of accordance with the DASH diet pattern. The analytical sample was 54% female and the mean age was 59 years. The mean (total in brackets) length of follow up was 12.0 person-years (282,952) for incident IHD, 12.4 person-years (294,323) for incident stroke, 12.4 person-years (292,822) for incident CVD, and 17.1 person-years (405,181) for all deaths.

Table 2 reports the demographic, behavioral and health characteristics of participants, as measured at baseline, across quintiles of DASH accordance. There was apparent patterning of many sociodemographic, behavioural and health characteristics across quintiles, including known risk factors for CVD. Intermediate clinical cardiovascular risk factors including blood pressure and lipids also showed patterning across the quintiles of DASH accordance.

**Table 2:** Characteristics of the analytical sample (n = 23,655; EPIC-Norfolk Cohort)

	Quintile of accordance to DASH				
	1 Least accordant	2	3	4	5 Most accordant
Number	5744	5227	3703	4800	4181
*Sociodemographic, behavioral and medical risk factors*					
Mean age (years)	59.7	59.4	58.9	59.1	58.0
% Male	58	50	44	40	33
% Current smoker	19	12	10	8	6
% Overweight and obese	62	62	62	59	55
% Inactive^a^	34	32	31	28	22
Mean weekly units of alcohol	7.2	7.4	7.2	7.3	6.7
Semi- and non-skilled occupational social class^b^	22	16	16	14	13
% Single	4.1	3.6	3.8	3.7	4.1
% Diabetes^c^	1.5	1.7	2.8	2.7	2.9
% Anti-hypertensive medication^d^	17	19	20	19	18
% Lipid lowering medication^e^	1.0	1.4	1.6	1.5	2.3
% History of CVD^f^	4.5	4.7	4.1	4.0	4.4
*Intermediate risk factors*					
Systolic blood pressure (mmHg)	136.8	136.1	134.7	135.0	133.5
Diastolic blood pressure (mmHg)	83.4	82.8	82.2	82.2	81.5
Total cholesterol (mmol/L)	6.23	6.19	6.18	6.19	6.07
HDL cholesterol (mmol/L)	1.35	1.39	1.42	1.45	1.49
LDL cholesterol (mmol/L)	4.02	3.98	3.97	3.97	3.84
*Outcomes*					
Number of incident IHD events	975	818	520	624	494
Number of incident stroke events	318	262	157	209	150
Number of CVD deaths	479	423	264	289	192

^a^Lowest category of physical activity; ^b^lowest two occupational social groups, based on the Registrar General classification; ^c^self-reported past diagnosis of diabetes; ^d^use of any medications indicated for hypertension; ^e^use of any medications indicated for high cholesterol or dislipidemia; ^f^recorded cardiovascular disease event

Incident stroke and incident CVD were negatively associated with accordance to the DASH diet pattern (note—both fatal and non-fatal events were included for all incident outcomes). Table 3 contains the hazard ratios and 95% confidence intervals for an association between quintile of DASH accordance and incident IHD, incident stroke and incident CVD, with the lowest quintile of DASH accordance as the reference category. When adjustment was made for only age, sex and total dietary energy an association was found between DASH accordance and incident IHD (HR_Q5_ 0.87 95% CI 0.77, 0.99), incident stroke (HR_Q5_ 0.79 95% CI 0.64, 0.97) and incident CVD (HR_Q5_ 0.85 95% CI 0.76, 0.95). After adjustment for further sources of confounding these associations were attenuated but a significant association remained between the quintile of greatest DASH accordance and both incident stroke (HR_Q5_ 0.80 95% CI 0.65, 0.99) and incident CVD (HR_Q5_ 0.88 95% CI 0.79, 0.99). A significant association was found between incident IHD and the middle quintile of DASH accordance (HR_Q3_ 0.88 95% CI 0.78, 0.99).

**Table 3:** Hazard ratios and 95% confidence intervals for incident ischaemic heart disease, incident cerebrovascular disease and incident cardiovascular disease, by quintile of DASH accordance score; (EPIC-Norfolk cohort, n = 23,655)

Quintile of accordance to DASH	Events per 100,000 person-years	Model 1^a^		Model 2^b^	
*Incident IHD*					
Q1	1262.2	1.00	(Ref.)	1.00	(Ref.)
Q2	1125.0	0.95	(0.86–1.05)	0.95	(0.86–1.05)
Q3	982.6	0.88*	(0.79–0.99)	0.88*	(0.78–0.99)
Q4	916.2	0.85**	(0.76–0.95)	0.91	(0.81–1.01)
Q5	799.7	0.87*	(0.77–0.99)	0.90	(0.79–1.02)
*Incident stroke*					
Q1	422.0	1.00	(Ref.)	1.00	(Ref.)
Q2	376.9	0.93	(0.78–1.10)	0.95	(0.80–1.12)
Q3	323.3	0.84	(0.69–1.02)	0.85	(0.69–1.04)
Q4	320.5	0.84	(0.70–1.01)	0.87	(0.73–1.05)
Q5	261.3	0.79*	(0.64–0.97)	0.80*	(0.65–0.99)
*Incident CVD*					
Q1	1471.7	1.00	(Ref.)	1.00	(Ref.)
Q2	1304.6	0.94	(0.86–1.03)	0.95	(0.87–1.04)
Q3	1154.2	0.88*	(0.79–0.98)	0.89*	(0.80–0.99)
Q4	1071.2	0.85**	(0.77–0.94)	0.90*	(0.81–0.99)
Q5	929.5	0.85**	(0.76–0.95)	0.88*	(0.79–0.99)

*p < 0.05; **p < 0.01;***p < 0.001

^a^Adjusted for age, sex and dietary energy

^b^As Model 1 but further adjusted for smoking status, alcohol intake, physical activity, BMI, diabetes, SES, marital status, use of antihypertensive medication, use of lipid-lowering medication and history of CVD

CVD-specific mortality and all-cause mortality were negatively associated with accordance with the DASH diet pattern. Table 4 contains the hazard ratios and 95% confidence intervals for an association between quintile of DASH accordance and CVD mortality, non-CVD mortality and all-cause mortality, with the lowest quintile of DASH accordance as the reference category. When age, sex and total dietary energy were the only covariates included in the model, greater DASH accordance was associated with lower risk of CVD mortality (HR_Q5_ 0.75 95% CI 0.63, 0.88), non-CVD mortality (HR_Q5_ 0.86 95% CI 0.78, 0.94), and all-cause mortality (HR_Q5_ 0.83 95% CI 0.77, 0.90). Once all the other covariates were included these relationships were attenuated so that the quintile of greatest DASH accordance was associated with only CVD mortality (HR_Q5_ 0.72 95% CI 0.60, 0.85) and all-cause mortality (HR_Q5_ 0.87 95% CI 0.80, 0.95). However, the middle quintile of DASH accordance remained associated with non-CVD mortality (HR_Q3_ 0.90 95% CI 0.82, 0.99).

**Table 4:** Hazard ratios and 95% confidence intervals for CVD mortality, non-CVD mortality and all-cause mortality, by quintile of DASH accordance score, (EPIC-Norfolk cohort, n = 23,655)

Quintile of accordance to DASH	Events per 100,000 person-years	Model 1^a^		Model 2^b^	
*Cardiovascular mortality*					
Q1	495.3	1.00	(Ref.)	1.00	(Ref.)
Q2	478.1	1.04	(0.91–1.18)	1.05	(0.92–1.20)
Q3	414.6	0.95	(0.81–1.10)	0.95	(0.81–1.10)
Q4	348.5	0.82**	(0.71–0.95)	0.84*	(0.72–0.98)
Q5	261.6	0.75**	(0.63–0.88)	0.72***	(0.60–0.85)
*Non-cardiovascular disease mortality*					
Q1	1444.7	1.00	(Ref.)	1.00	(Ref.)
Q2	1300.9	0.95	(0.88–1.03)	0.99	(0.91–1.07)
Q3	1133.9	0.86**	(0.79–0.94)	0.90*	(0.82–0.99)
Q4	1121.4	0.86***	(0.79–0.93)	0.93	(0.85–1.01)
Q5	976.9	0.86**	(0.78–0.94)	0.93	(0.85–1.02)
*All-cause mortality*					
Q1	1940.0	1.00	(Ref.)	1.00	(Ref.)
Q2	1779.0	0.97	(0.91–1.04)	1.01	(0.94–1.08)
Q3	1548.5	0.88**	(0.82–0.95)	0.91*	(0.84–0.99)
Q4	1471.0	0.85***	(0.79–0.91)	0.91**	(0.84–0.98)
Q5	1238.5	0.83***	(0.77–0.90)	0.87**	(0.80–0.95)

*p < 0.05;**p < 0.01;***p < 0.001

^a^Adjusted for age, sex and dietary energy

^b^As Model 1 but further adjusted for smoking status, alcohol intake, physical activity, BMI, diabetes, SES, marital status, use of antihypertensive medication, use of lipid-lowering medication and history of CVD

Sensitivity analyses revealed that adding total serum cholesterol and systolic blood pressure to Model 2 led to there being no significant association between DASH accordance and any incident outcome, whilst similar associations remained between the most accordant DASH quintile and both CVD mortality and total mortality. Including DASH accordance as a continuous (rather than categorical) variable to Model 2 resulted in a significant inverse relationship between DASH accordance and incident stroke, CVD mortality, non-CVD mortality and all-cause mortality, but not incident IHD or incident CVD.

## Discussion

This study investigated the association between accordance to the DASH dietary pattern and both incident CVD events and CVD mortality using data from 23,655 people in the EPIC-Norfolk cohort. In adjusted models there was a significant association—indicating lower risk—between greater DASH accordance and incident stroke and incident CVD, but not incident IHD. Greater DASH accordance was also associated with lower risk of total CVD mortality and all-cause mortality, although not between greater DASH accordance and non-CVD mortality.

### Interpretation and comparison with other studies

The association observed here between accordance to the DASH diet and incident CVD is in line with expectations, with a meta-analysis showing a significant association for this relationship (RR 0.80 95% CI 0.74, 0.86), in samples from the USA, Italy and Sweden [17]. The estimate of a 12% lower risk found here is smaller than the 20% reduction found in that meta-analysis but the majority of studies used in the meta-analysis only contained women, and several studies were based on occupational cohorts, which is likely to reduce the absolute risk of CVD in the sample when compared to a population-based sample of older men and women like the one used here. The dominant study in the meta-analysis was completed by Fung et al. based on an occupational cohort of women from the USA. The absolute risk of incident IHD observed in the lowest quintile of DASH accordance in that cohort was 165 cases per 100,000 person-years, in contrast to 1262 cases per 100,000 person-years in the present study [19]. The greater overall risk in this cohort may result in an apparent weaker association between CVD and dietary risk factors. Rothman’s Sufficient-Component Cause model [35] stated that a given disease outcome may have multiple causes that are each sufficient to cause disease, with only some being necessary. Since dietary risk factors are not a necessary cause for CVD events, a high level of CVD risk in this sample may reflect a lower relative contribution of diet compared to other risk factors (particularly age-related factors), making the association between this measure of diet and CVD less detectable. Given this, the difference in sample characteristics may explain the difference in the magnitude of the association between that found by Salehi-Abargouei et al. and the present study.

This meta-analysis of Salehi-Abargouei et al. did not report the same distinction between IHD and stroke as shown here, with the pooled results showing a significant reduction in risk for both incident Stroke and incident IHD. That meta-analysis was pooled from three studies, of which only one found a significant association and this was conducted by Fung et al. [19]. The Fung et al. study was much larger (n = 88,517) than either the present study or the two others included in the meta-analysis, which may have been the cause of reduced uncertainty around the estimate and the significant result they report for that outcome. However, the lower risk of IHD associated with DASH observed here was only 10%, less than the 21% observed in the meta-analysis, so both the scale of the reduction and lack of significance is at odds with that observed there.

The finding that increased accordance to the DASH diet is associated with CVD mortality is in accordance with the findings of previous studies, all of which used samples from the USA [21, 36, 37]. Given the concordance between these studies and the present findings, both in direction and magnitude of the association observed, it is probable that the results presented here are true findings. Furthermore, the specificity of the association between DASH accordance and CVD mortality (but not non-CVD mortality) suggests biological plausibility and reduces our concern that these associations were the result of residual confounding by unmeasured social, behavioural or clinical risk factors.

The association observed between the third quintile of DASH accordance and both incident IHD and non-CVD mortality, yet not any other quintile of DASH accordance with these outcomes, is harder to interpret. It is possible that these are chance findings or that there are unobserved factors associated with these outcomes and the third quintile of DASH accordance to the exclusion of the other quintiles. By comparison, the associations between DASH and stroke and CVD-related mortality followed an apparent dose–response pattern, with largest associations observed in the contrast between the least and most DASH-accordant quintiles, these can reasonably be interpreted as being likely to be due to the composition of the DASH diet.

### Limitations and methodological considerations

The main limitation of this study stems from the use of self-reported dietary data. This method is subjective and vulnerable to both random and systematic error, with the potential to lead to misclassification bias [38]. This could result from random error, which would bias the results towards the null and attenuate any real association, or it could be driven by social desirability bias, where intakes of healthier foods are overstated while intakes of less-healthy foods are under-reported [39]. However, with no knowledge of how social desirability is distributed across the sample, it is not possible to meaningfully comment on the direction or magnitude that this may have on the results reported here. It is plausible that obese participants were more likely to selectively underreport dietary intakes, as this has been shown in previous studies [40], which could have led to differential misclassification of DASH accordance based on obesity. However, it should be noted that limitations of this nature affect all subjective methods of dietary assessment, so this should not limit the comparability of these results to those of other studies. We excluded approximately 7% of the sample, mainly due to lack of dietary or covariate information. The excluded participants had characteristics associated with elevated CVD risk: older, more likely to smoke, and with higher blood pressure. This exclusion likely biased our results in a way that attenuated the associations we observed, since these individuals would have likely consumed less DASH-accordant diets and would have been at higher risk for CVD. Although the cohort design allows the observation of a temporal relationship between the exposure and outcome, it is still an observational design and is therefore susceptible to residual confounding, although the direction or magnitude of any such effect is not known.

This study has a number of strengths, including its large sample size, prospective design, basis in the general population, independent assessment of outcome events, and a broad range of available factors to include as covariates. The ascertainment of dietary exposures and covariate data at the start of the study eliminates potential recall bias that would affect retrospective study designs. The use of administrative records to identify outcomes minimizes loss to follow-up and ascertainment bias. To our knowledge, this is the first prospective study to examine the association between DASH accordance and CVD in the UK.

### Generalisability

The generalisability of these results may be affected by the age of the study sample, which is older than the general UK population. However, CVD events are more likely to occur in people aged over 40 years, meaning that this sample represents an important population in which to assess the effectiveness of a diet intended to reduce the risk of CVD. Adjustment for possible confounding factors means that the results presented here can be compared to those of cohorts with a different distribution of these factors, allowing for its inclusion in future meta-analyses. As stated previously, at the time the EPIC-Norfolk study began, this cohort was similar to the wider UK population in terms of key risk factors, including height, weight, blood pressure and total cholesterol, which lends support to the external validity of the results presented here.

### Implications for public health

The epidemiological evidence presented here adds to a well-established literature on the importance of diet for prevention of chronic disease. In both the UK and USA, dietary recommendations are part of government strategy for population-level health promotion. However the uptake of dietary guidance and the adoption of healthier eating habits in the population may be hindered by a number of behavioral and social factors. Studies have suggested that healthy eating depends on food literacy and nutrition knowledge [41], cooking [42, 43], and the availability of time to spend in food preparation [44], and the eating habits of family, friends and peers [45, 46]. The cost of food and neighborhood food access may also be important in particular for the DASH diet. Research in the UK and USA indicate that DASH accordant diets tend to cost more than less-healthy diets [31, 47, 48] and studies from the UK and Ireland have found that living in closer proximity to supermarkets may enable dietary habits that are congruent with the DASH pattern [48, 49]. Given the balance of evidence, the promotion of DASH and other cardioprotective diets needs to be matched with both population- and individual-level interventions to support the uptake of healthy eating.

## Conclusion

This study has shown that the DASH diet pattern is associated with significantly lower risk of incident stroke, incident CVD and CVD mortality, consistent with previous studies conducted mainly in the USA. The results presented here indicate that the DASH dietary pattern does have relevance for the UK population and can be used as a salient measure of a cardioprotective diet. As such it could have a role in future dietary public health promotion strategies.

## Abbreviations

CVD: Cardiovascular disease
DASH: Dietary approaches to stop hypertension
FFQ: Food frequency questionnaire
IHD: Ischaemic heart disease
SES: Socioeconomic status
UK: United Kingdom
